# Clinical outcome implications of novel auscultation device of artificial intelligence (NAVI-HF) in predischarge acute heart failure patients

**DOI:** 10.3389/fcvm.2026.1864004

**Published:** 2026-10-27

**Authors:** Rony Marethianto Santoso, Ser Wee, Amiliana Mardiani Soesanto, Wisnu Jatmiko, Aria Kekalih, Nuri Dyah Indrasari, Yong Kiang Yeo, Wilbert Huang, Bambang Budi Siswanto

**Affiliations:** 1 Doctoral Program in Medical Sciences, Faculty of Medicine, University of Indonesia, Jakarta, Indonesia; 2 Primaya Heart and Vascular Center Hospital, Tangerang, Indonesia; 3 School of Electrical and Electronic Engineering, Nanyang Technology University, Singapore, Singapore; 4 SPACE (MedTech) Pte. Ltd., Singapore, Singapore; 5 National Cardiovascular Center Harapan Kita, Jakarta, Indonesia; 6 Department of Cardiology and Vascular Medicine, Faculty of Medicine, University of Indonesia, Jakarta, Indonesia; 7 Faculty of Computer Science, University of Indonesia, Jakarta, Indonesia; 8 Department of Community Medicine, Faculty of Medicine, University of Indonesia, Jakarta, Indonesia; 9 Department of Clinical Pathology, Faculty of Medicine, University of Indonesia, Jakarta, Indonesia

**Keywords:** artificial intelligence, heart failure, lung ultrasound, machine learning, prognosis

## Abstract

**Aim:**

This study evaluated the prognostic significance of a novel auscultation device of artificial intelligence (NAVI-HF) in predicting clinical outcomes in predischarge acute heart failure (HF) patients.

**Methods:**

This second, prognostic phase of a prospective observational cohort study extends our prior diagnostics phase, in which NAVI-HF demonstrated good performance compared with lung ultrasound (LUS) B-lines as the reference standard. Patients were classified as NAVI-HF positive or negative prior to discharge and followed for 6 months for evaluation.

**Results:**

A total of 239 patients were included of whom 111 (45.1%) were NAVI-HF positive. NAVI-HF positive patients had a worse clinical profile and higher NT-proBNP (*p* < 0.05). During a median follow-up of 6 months, 82 composite events (34.3%) occurred. In univariate Cox analysis, NAVI-HF positive was associated with significant increased risk of composite outcome (HR 1.59, CI 1.03–2.47; p 0.03) and HF hospitalization (HR 1.64, CI 1.05–2.46; p 0.02), but not all-cause mortality (p 0.24). The association in composite and HF hospitalization outcomes remained significant after adjustment for age and sex but was attenuated after adjustment for other prognostic variables.

**Conclusion:**

NAVI-HF–detected congestion was associated with increased adverse clinical outcomes in predischarge acute HF patients.

## 1 Introduction

Heart failure (HF) remains an important health burden worldwide, with recurrent congestion being the primary driver of clinical deterioration and readmission (1). Despite advances in guideline-directed therapy, many patients are discharged with residual or subclinical congestion that may not be apparent on routine clinical examination and those patients were also associated with worse outcome (2). This limitation highlights the ongoing challenge of accurately assessing volume status and identifying patients at risk for early adverse outcomes.

Objective tools to evaluate congestion have therefore become increasingly important. Biomarkers such as NT-proBNP provide insight into myocardial wall stress, while lung ultrasound (LUS) allows direct visualization of pulmonary congestion through B-lines (3–6). Prior studies have demonstrated the diagnostic and prognostic value of LUS in both acute and ambulatory HF settings, and residual B-lines have been consistently associated with worse clinical outcomes (3). However, the implementation of LUS in routine and home-based care remains limited by the need for operator expertise and equipment availability.

To address this gap, we developed NAVI-HF as a simplified, novel auscultation tool aided with artificial intelligence designed to detect pulmonary congestion for home-based monitoring in heart failure patients. In the first phase of the NAVI-HF study, we demonstrated promising diagnostic performance compared with LUS-defined B-lines, establishing its feasibility as a non-invasive congestion assessment method.

The present study represents the second phase of this investigation. Rather than focusing on diagnostic accuracy, we aimed to evaluate the clinical outcome implications of NAVI-HF–detected congestion (NAVI- HF positive). Specifically, we assessed whether congestion identified by NAVI-HF in predischarge HF patients is associated with subsequent clinical outcomes. By moving from diagnostic validation to prognostic evaluation, this phase seeks to determine whether NAVI-HF captures a congestion profile that is not only measurable, but clinically meaningful.

## 2 Methods

### 2.1 Study design and population

This study represents the second phase of a multicenter, prospective, observational cohort study designed to evaluate the prognostic significance of NAVI-HF in predischarge patients with acute heart failure and reduced ejection fraction (HFrEF). In brief, NAVI-HF is a novel auscultation device aided with artificial intelligence with a stethoscope-like interface designed to capture and record thoracic cavity sound signals, including both pulmonary and cardiac sounds. Through advanced signal processing, the device enables detection of acoustic signatures associated with underlying cardiopulmonary abnormalities – pulmonary congestion. The initial phase investigated the diagnostic performance of NAVI-HF in detecting pulmonary congestion, using lung ultrasound (LUS) B-lines as the reference standard. The study demonstrated promising diagnostic accuracy, including the ability to detect subclinical congestion with an accuracy of 98% and 86% in the training and testing set, respectively. Patients identified to have pulmonary congestion by NAVI-HF positive is regarded as NAVI-HF positive and those without pulmonary congestion is regarded as NAVI-HF negative. A detailed description of the NAVI-HF device, including its development and diagnostic performance, is provided in Supplementary 1.

The present phase extends these findings through a translational approach, assessing whether NAVI-HF–detected congestion is associated with subsequent clinical outcomes. Patients were followed for 6 months after discharge to evaluate the occurrence of composite outcomes, defined as all-cause mortality and HF hospitalization.

Sample size was calculated assuming a hazard ratio (HR) of 2.63 for adverse clinical outcomes in patients with positive lung congestion on LUS (2), and an HF rehospitalization rate of 30% in Southeast Asian countries based on the REPORT-HF study (7).

The Institutional Review Boards of National Cardiac Center Harapan Kita Hospital (DP.04.03/KEP011/EC003/2025), University of Indonesia – Cipto Mangunkusumo Hospital (KET-300/UN2.F1/ETIK/PPM.00.02/2025), and Rumah Sakit Primaya Tangerang (005/KOMED/PHTA/I/2025) approved this study protocol and inform consent was obtained from the patients enrolled in the study. This manuscript adheres to the STROBE (Strengthening the Reporting of Observational Studies in Epidemiology) guidelines (8).

### 2.3 Data collection and outcome definition

Baseline patient characteristics were collected as described in the previous phase of the study. For this follow-up phase, clinical outcomes were assessed at 6 months post-discharge. The primary endpoint was a composite of all-cause mortality and heart failure (HF) hospitalization. In addition to the composite endpoint, each component was also analyzed separately as secondary outcomes.

All-cause mortality was ascertained through review of electronic medical records. In cases of incomplete documentation, structured telephone follow-up was conducted to confirm vital status and minimize loss to follow-up.

HF hospitalization was defined as an unplanned hospital admission primarily attributable to worsening signs and/or symptoms of heart failure requiring inpatient management.

### 2.5 Statistical analysis

All statistical analyses were performed using IBM SPSS Statistics version 26.0. A two-sided p-value <0.05 was considered statistically significant.

Continuous variables are presented as mean ± standard deviation or median (Q1 and Q3), as appropriate based on distribution, and were compared using the independent t-test or Mann–Whitney U test. Categorical variables are expressed as frequencies and percentages and were compared using the *χ*^2^ test or Fisher's exact test, as appropriate.

Time-to-event analyses were calculated in months from the date of hospital discharge to the occurrence of the first evet or censoring. Patients were censored at the time of last follow-up if no event occurred within the 6-month observation period. Analysis was performed using the Kaplan–Meier method to estimate cumulative event rates and displayed with survival function for the composite outcome of all-cause mortality and HF hospitalization. Differences between groups were assessed using the log-rank (Mantel–Cox) test. The association between NAVI-HF findings and clinical outcomes was evaluated using Cox proportional hazards regression models. Hazard ratios (HRs) with 95% confidence intervals (CIs) were reported. The proportional hazards assumption was assessed using Schoenfeld residuals and log-minus-log survival plots.

Multivariable Cox regression analyses were constructed using a stepwise modelling approach with four predefined models to account for potential confounding. Covariates were selected based on clinical relevance and established prognostic significance in heart failure. The number of covariates included in each model was determined according to the rule-of-thumb principle of a minimum of 10 outcome events per variable to reduce the risk of overfitting and preserve model stability.

Model 1 comprised the unadjusted analysis evaluating the association between NAVI-HF findings and outcomes. Model 2 adjusted for demographic variables, including age and sex. Model 3 further adjusted for established prognostic factors in heart failure, namely hypertension, diabetes mellitus, chronic kidney disease, NT-pro BNP (per 100 pg/mL increment), and left ventricular ejection fraction (LVEF; per 10% increment). Additional model by incorporating other prognostic variables such as coronary artery disease, use of guideline directed medical therapies, echocardiographic parameters, log transformation of NT-proBNP were also conducted in sensitivity analysis.

## 3 Results

### 3.1 Patient selection and characteristics

Initially, 246 samples were collected from four participating centres. Seven samples were excluded using an entropy-based outlier detection algorithm, resulting in 239 samples included in the final analysis. Unlike the previous phase, which excluded patients with missing lung ultrasound (LUS) data, this phase adopted a more inclusive strategy by enrolling all patients who underwent NAVI-HF examination regardless of LUS availability **(**Figure 1**)**.

**Figure 1:**
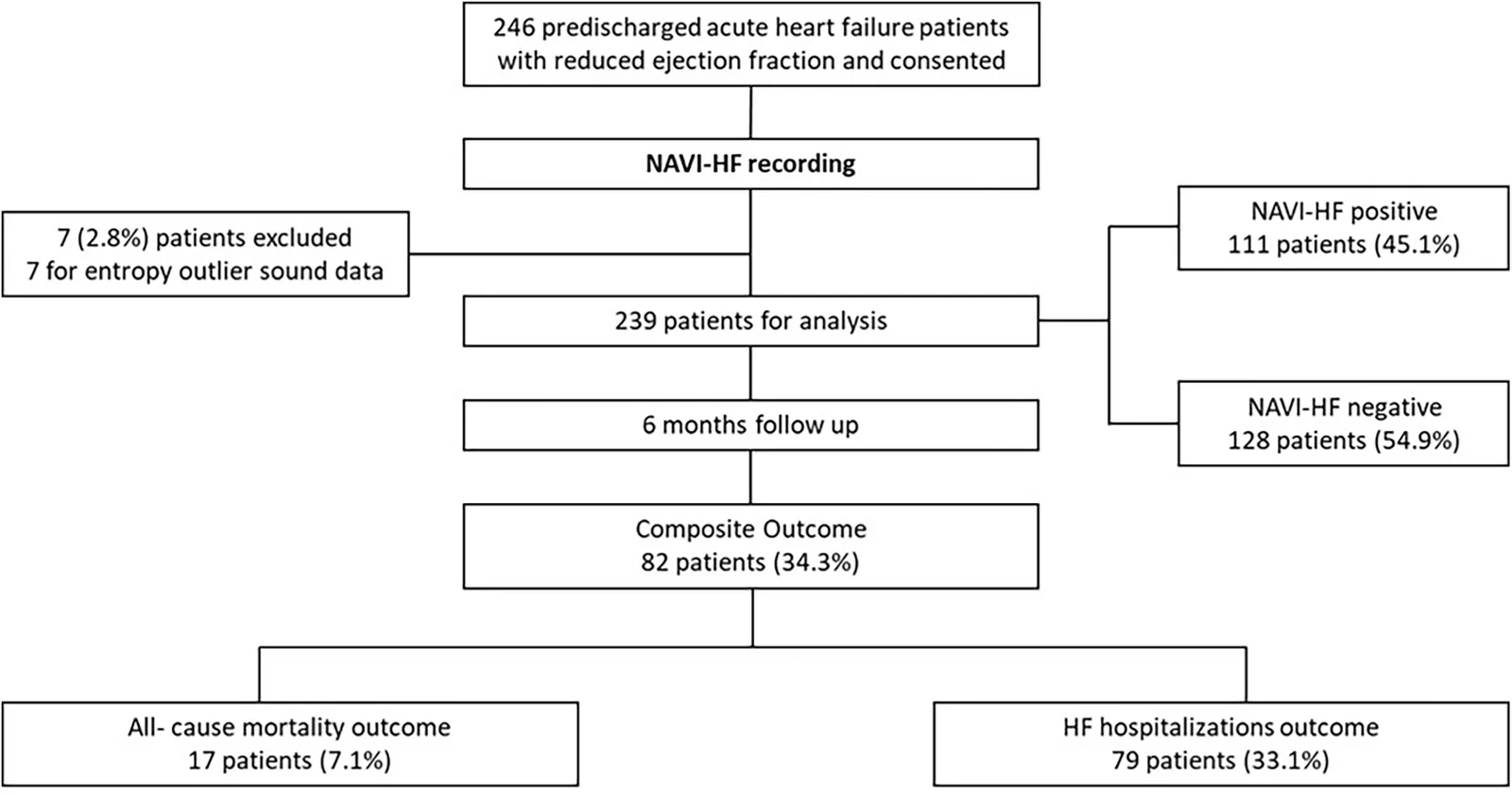
Workflow of second phase NAVI-HF study.

The mean age of the study population was 57.5 ± 10.7 years, and 75.3% were male. The median systolic and diastolic blood pressures at discharge were 120 mmHg and 80 mmHg, respectively. Comorbidities were common, including hypertension (63.2%), diabetes mellitus (44.4%), coronary artery disease (78.7%), and chronic kidney disease (23.8%). Most patients were classified as New York Heart Association (NYHA) functional class II (59.1%) during the predischarge period. *De novo* heart failure accounted for 28.0% of cases, while the remainder had a prior history of heart failure hospitalization.

The median left ventricular ejection fraction (LVEF) was 34% (IQR 25–39), and the median NT-pro BNP level was 2923.5 pg/mL (IQR 951–8420). At discharge, only 25.5% of patients had pulmonary crackles on physical examination, and 12.6% had extremities edema. Lung ultrasound B- lines was positive in 47.3% of patients.

Loop diuretics were the most frequently prescribed therapy, followed by renin–angiotensin system inhibitors (ACE inhibitors, ARBs, or ARNIs), mineralocorticoid receptor antagonists (MRAs), beta-blockers, and sodium–glucose cotransporter-2 (SGLT2) inhibitors **(**Table 1**)**.

**Table 1:** Patient baseline characteristics.

Baseline characteristics	NAVI HF (+) *n* = 111	NAVI HF (-) *n* = 128	Total *n* = 239	*p* value
**Gender,** n (%)				
Male	81 (73.0)	99 (77.3)	180 (75.3)	0.45
Female	30 (27.0)	29 (22.7)	59 (24.7)	
**Age (years),** mean (SD)	58.4 (10.7)	56.9 (10.8)	57.5 (10.7)	0.15
**BMI (kg/m2),** median (Q1 - Q3)	24.3 (21.5 - 27.4)	24.8 (22.8 - 27.2)	24.5 (22.2, 27.3)	0.34
**Hypertension,** n (%)	79 (71.2)	72 (56.3)	151 (63.2)	**0**.**02**
**Diabetes Mellitus,** n (%)	58 (52.3)	48 (37.5)	106 (44.4)	**0**.**03**
**Dyslipidemia,** n (%)	41 (36.9)	41 (32.0)	82 (34.3)	0.49
**Stroke,** n (%)	10 (9.0)	7 (5.5)	17 (7.1)	0.32
**Chronic kidney disease,** n (%)	35 (31.5)	22 (17.2)	57 (23.8)	**0**.**01**
**Coronary artery disease,** n (%)	84 (75.7)	104 (81.3)	188 (78.7)	0.34
**Atrial fibrillation,** n (%)	11 (9.9)	13 (10.2)	24 (10.0)	0.99
**Smoking**				
Active	6 (5.4)	9 (7.0)	15 (6.3)	0.87
Stopped	73 (65.8)	82 (64.1)	155 (64.9)	
**Number of prior HF hospitalizations,** n (%)				
0	30 (27.0)	37 (28.9)	67 (28.0)	0.57
1	38 (34.2)	45 (35.2)	83 (34.7)	
2	14 (12.6)	18 (14.1)	32 (13.4)	
>3	29 (26.1)	28 (21.9)	57 (23.8)	
**Systolic BP (mmHg),** median (Q1 - Q3)	120.0 (109.8, 130.0)	120.0 (110.0, 120.0)	120.0 (110.0, 120.0)	**<0** **.** **001**
**Diastolic BP (mmHg),** median (Q1 - Q3)	80.0 (70.0, 80.0)	80.0 (70.0, 80.0)	80.0 (70.0, 80.0)	**<0** **.** **001**
**Heart Rate (bpm),** median (Q1 - Q3)	81.0 (74.3, 90.5)	78.0 (74.0, 85.0)	79.0 (74.0, 87.0)	**0**.**013**
**Crackles,** n (%)	57 (51.4)	4 (3.1)	61 (25.5)	**<0** **.** **001**
**Extremities edema,** n (%)	24 (21.6)	6 (4.7)	30 (12.6)	**<0** **.** **001**
**NYHA functional class,** n (%)				
I	17 (15.3)	45 (35.7)	62 (26.2)	**<0** **.** **001**
II	65 (58.6)	75 (59.5)	140 (59.1)	
III	24 (21.6)	6 (4.8)	30 (12.7)	
IV	5 (4.5)	0 (0.0)	5 (2.1)	
**Lung ultrasound**	**(*n*** **=** **111)**	**(*n*** **=** **113)**	**(*n*** **=** **224)**	
Positive	104 (93.7)	2 (1.8)	106 (47.3)	**<0** **.** **001**
Negative	7 (6.3)	111 (98.2)	118 (52.7)	
**NT-pro BNP (pg/mL),** median (Q1 - Q3)	7523.5 (2433.3, 17179.5)	1964.0 (671.8, 4490.3)	2923.5 (951.0, 8420.0)	**<0** **.** **001**
**LVEF (%),** median (Q1 - Q3)	34.0 (23.0, 39.0)	34.0 (26.0, 39.0)	34.0 (25.0, 39.0)	0.073
**LA diameter (mm),** median (Q1 - Q3)	4.2 (3.7, 4.6)	4.1 (3.5, 4.4)	4.1 (3.6, 4.5)	0.105
**LA volume index (%),** median (Q1 - Q3)	44.9 (36.0, 56.2)	39.3 (30.4, 48.3)	40.7 (31.9, 51.2)	0.107
**LVEDV (mL),** median (Q1 - Q3)	180.0 (151.3, 236.0)	184.0 (145.5, 213.5)	182.5 (149.0, 224.0)	0.349
**LVESV (mL),** median (Q1 - Q3)	119.5 (93.6, 173.5)	120.0 (98.0, 157.0)	119.5 (98.0, 161.3)	0.228
**E/ A**	1.6 (0.9, 2.3)	1.4 (0.7, 2.5)	1.5 (0.7, 2.4)	0.09
**E/ E'**	21.0 (13.1, 31.1)	19.9 (14.2, 26.4)	20.0 (13.9, 27.7)	0.78
	**(*n*** **=** **78)**	**(*n*** **=** **102)**	**(*n*** **=** **180)**	
**IVC distensibility,** n (%)	45 (57.7)	67 (65.7)	112 (62.2)	0.28
**Mitral Regurgitation,** n (%)	**(*n*** **=** **110)**	**(*n*** **=** **127)**	**(*n*** **=** **227)**	
None	14 (12.7)	21 (16.5)	35 (14.8)	0.15
Mild	60 (54.5)	73 (57.5)	133 (56.1)	
Moderate	19 (17.3)	22 (17.3)	41 (17.3)	
Severe	17 (15.5)	11 (8.7)	28 (11.8)	
**Tricuspid Regurgitation,** n (%)				
None	41 (37.3)	68 (53.5)	109 (46.0)	**< 0** **.** **001**
Mild	41 (37.3)	48 (37.8)	89 (37.6)	
Moderate	19 (17.3)	7 (5.5)	26 (11.0)	
Severe	9 (8.2)	4 (3.1)	13 (5.5)	
**Aortic Regurgitation,** n (%)				
None	63 (57.3)	80 (63.0)	143 (60.3)	0.34
Mild	85 (35.9)	43 (33.9)	85 (35.9)	
Moderate	8 (3.4)	4 (3.1)	8 (3.4)	
Severe	1 (0.4)	0 (0.0)	1 (0.4)	
**Pulmonary Regurgitation,** n (%)				
None	85 (77.3)	114 (89.8)	199 (84.0)	**<0** **.** **009**
Mild	22 (20.0)	12 (9.4)	34 (14.3)	
Moderate	2 (1.8)	1 (0.8)	3 (1.3)	
Severe	1 (0.9)	0 (0.0)	1 (0.4)	
**Loop diuretics,** n (%)	100 (90.1)	103 (80.5)	203 (84.9)	**0**.**04**
**Beta blockers,** n (%)	69 (62.2)	85 (66.4)	154 (64.4)	0.5
**ACE inhibitors/ ARB,** n (%)	74 (66.7)	70 (54.7)	144 (60.3)	0.06
**ARNI,** n (%)	29 (26.1)	51 (39.8)	80 (33.5)	**0**.**02**
**Mineralocorticoid receptor antagonist,** n (%)	83 (74.8)	75 (58.6)	158 (66.1)	**0**.**009**
**SGLT2 inhibitors,** n (%)	7 (6.3)	10 (7.8)	17 (7.1)	0.8

*p-value between NAVI- HF group.

Statistical significant values are expressed in bold.

### 3.2 Comparison between NAVI-HF positive and negative groups

There were 111 patients (45.1%) with NAVI- HF positive and 128 patients (54.9%) with NAVI-HF negative. Patients with positive NAVI-HF findings had a higher prevalence of hypertension (*p* = 0.02), diabetes mellitus (*p* = 0.03), and chronic kidney disease (*p* = 0.01). They also exhibited higher systolic and diastolic blood pressures and elevated heart rates at discharge (all *p* < 0.05).

Clinically, the NAVI-HF positive group had a greater proportion of patients in NYHA functional class III at discharge (*p* < 0.001), a higher prevalence of positive LUS findings (*p* < 0.001), and significantly elevated NT-pro BNP levels (*p* < 0.001). Echocardiographic assessment revealed a higher prevalence of tricuspid and pulmonary regurgitation in this group (*p* < 0.001).

Regarding pharmacotherapy, patients with positive NAVI-HF findings were more frequently treated with ARNIs (*p* = 0.02), loop diuretics (*p* = 0.046), and MRAs (*p* = 0.009) **(**Table 1**)**.

### 3.3 NAVI- HF and clinical outcomes

The median follow-up duration was 6 months. During follow-up, the composite outcome occurred in 82 patients (34.3%). All-cause mortality was observed in 17 patients (7.1%), and heart failure (HF) hospitalization occurred in 79 patients (33.1%) **(**Table 2**)**.

**Table 2:** Clinical outcomes in NAVI-HF study.

Clinical outcomes	NAVI HF (+) *n* = 111	NAVI HF (-) *n* = 128	Total *n* = 239
Composite outcome, n (%)	45 (40.5)	37 (28.9)	82 (34.3)
Time to event/ censor (months)	6.0 (3.0, 6.0)	6.0 (5.3, 6.0)	6.0 (4.7, 6.0)
All-cause mortality, n (%)	10 (9.0)	7 (5.5)	17 (7.1)
Time to event/ censor (months)	6.0 (6.0, 6.0)	6.0 (6.0, 6.0)	6.0 (6.0, 6.0)
HF rehospitalization, n (%)	44 (39.6)	35 (27.3)	79 (33.1)
Time to event/ censor (months)	6.0 (3.0, 6.0)	6.0 (5.3, 6.0)	6.0 (4.7, 6.0)

Kaplan–Meier analysis with Log-rank (Mantel–Cox) testing demonstrated that NAVI-HF positivity was significantly associated with a higher incidence of the composite outcome (*p* = 0.027) and HF hospitalization (*p* = 0.021), but not with all-cause mortality (*p* = 0.229) **(**Figure 2**)**. In unadjusted Cox proportional hazards analysis (Model 1), NAVI-HF positivity was associated with an increased risk of the composite outcome (HR 1.59; 95% CI: 1.03–2.47, *p* = 0.03) and HF hospitalization (HR 1.64; 95% CI: 1.05–2.56, *p* = 0.03), but not with all-cause mortality (*p* = 0.24). After adjustment for age and sex (Model 2), the associations remained statistically significant for both the composite outcome (HR 1.62; *p* = 0.03) and HF hospitalization (HR 1.68; *p* = 0.02). However, these associations were attenuated and no longer statistically significant (*p* = 0.60 and 0.50 for composite outcome and HF hospitalizations, respectively) after further adjustment for established prognostic factors, including hypertension, diabetes mellitus, chronic kidney disease, NT-pro BNP, and LVEF (Models 3) **(**Table 3**)**. Sensitivity analysis with other models did not alter the significance of the result (Supplementary 1).

**Figure 2:**
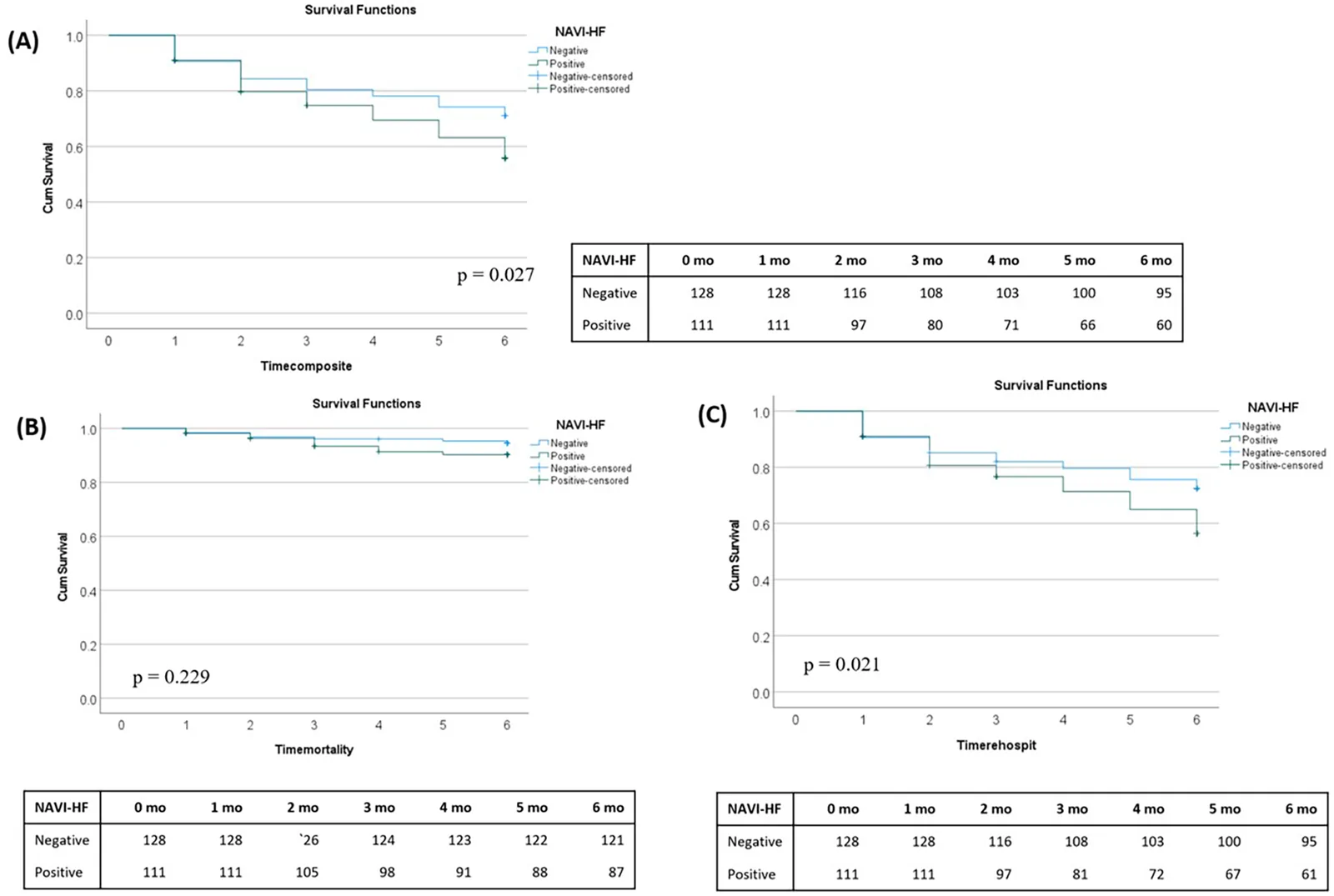
Kaplan–Meier survival curves according to NAVI-HF category for **(A)** composite outcome, **(B)** all-cause mortality, and **(C)** heart failure hospitalization.

**Table 3:** Univariate and multivariate cox regression analysis of NAVI-HF.

Variables	Composite Outcome			All- cause mortality			HF hospitalizations		
	Univariate analysis	Multivariate analysis		Univariate analysis	Multivariate analysis		Univariate analysis	Multivariate analysis	
	Model 1	Model 2	Model 3	Model 1	Model 2	Model 3	Model 1	Model 2	Model 3
NAVI- HF	1.59 (1.03–2.47)*	1.62 (1.05–2.51)*	1.13 (0.70–1.82)	1.79 (0.68–4.70)	1.76 (0.67–4.63)	1.19 (0.39–3.62)	1.64 (1.05–2.57)*	1.68 (1.07–2.63)*	1.18 (0.73–1.91)
Age (per year)	1.00 (0.98–1.02)	0.99 (0.97–1.02)	1.00 (0.98–1.02)	1.05 (1.00–1.10)*	1.06 (1.00–1.11)*	1.04 (0.99–1.09)	0.99 (0.97 - 1.01)	0.99 (0.97–1.01)	1.00 (0.98–1.02)
Gender (male)	0.79 (0.47–1.34)	0.77 (0.45–1.31)	0.76 (0.45–1.29)	0.63 (0.18–2.19)	0.48 (0.14–1.74)	0.46 (0.13–1.64)	0.77 (0.45–1.33)	0.76 (0.45–1.30)	0.77 (0.45–1.33)
Hypertension	1.19 (0.75–1.89)	-	1.12 (0.72–1.87)	1.42 (0.49–4.02)	-	1.02 (0.34–3.07)	1.33 (0.83–2.16)	-	1.27 (0.81–2.02)
Diabetes mellitus	1.39 (0.90–2.15)	-	1.34 (0.85–2.10)	1.79 (0.68–4.72)	-	1.37 (0.49–3.76)	1.30 (0.84–2.03)	-	1.13 (0.68–1.87)
Chronic kidney disease	1.37 (0.85–2.21)	-	1.08 (0.65–1.81)	1.35 (0.47–3.83)	-	0.75 (0.23–2.41)	1.37 (0.84–2.22)	-	1.12 (0.68–1.87)
NT-pro BNP (per 100 pg/mL)	1.002 (1.001–1.003)**	-	1.001 (1.000–1.002)*	1.003 (1.002–1.005)**	-	1.004 (1.001–1.006)*	1.002 (1.001–1.003)**	-	1.001 (1.000–1.002)*
LVEF (per 10%)	0.59 (0.46–0.77)**	-	0.63 (0.48–0.83)**	1.33 (0.70–2.49)	-	1.60 (0.77–3.32)	0.57 (0.44–0,75)**	-	0.60 (0.46–0.79)**

Model 2: adjusted for age and gender.

Model 3: adjusted for age, gender, hypertension, diabetes, chronic kidney disease, NT-pro BNP, LVEF.

*p value < 0.05.

**p value < 0.001.

## Discussion

The main findings of this study are as follows: (1) Patients classified as NAVI-HF positive were associated with a higher-risk clinical profile, characterized by a greater burden of comorbidities, more severe symptoms and signs of congestion, elevated congestion-related biomarkers, and higher use of decongestive therapies. (2) NAVI-HF–positive patients experienced worse composite clinical outcomes, primarily driven by HF hospitalizations. (3) The prognostic significance of NAVI-HF remained significant after adjustment for age and sex, but this association was attenuated after further adjustment for established prognostic variables. (4) NT-proBNP remained a strong and independent prognostic marker across all evaluated outcomes, including all-cause mortality.

This study represents the second phase of the NAVI-HF investigation. In the first phase (Supplementary 1), we demonstrated the promising diagnostic performance of NAVI-HF in detecting pulmonary congestion compared with lung ultrasound–derived B-lines, achieving an average accuracy of 98% in the training set and 86% in the testing set. In the present phase, we extended these findings to evaluate the prognostic significance of NAVI-HF with respect to clinically meaningful outcomes, including all-cause mortality and HF hospitalizations.

Patients classified as NAVI-HF positive exhibited a distinctly higher-risk clinical profile. They demonstrated more pronounced signs and symptoms of congestion, including a higher prevalence of pulmonary crackles, peripheral edema, and worse NYHA functional class. These patients also more frequently exhibited moderate-to-severe tricuspid and pulmonary regurgitation in echocardiography examinations, which may develop secondary to right-sided chamber enlargement and pulmonary hypertension in the setting of left-sided heart failure (9). Notably, moderate-to-severe tricuspid regurgitation has previously been associated with a 1.17–1.34-fold increased risk of mortality in HF patients, irrespective of the presence of left-sided valvular disease (10). Furthermore, NAVI-HF–positive patients were more frequently treated with higher-intensity decongestive therapies, likely reflecting more advanced or refractory congestion. This observation is consistent with prior data reported by Coiro et al. (11), who examined the relationship between LUS B-lines and hemodynamic indices of congestion. In that study, B-lines demonstrated good discriminatory ability for BNP levels >400 pg/mL and showed a moderate correlation with pulmonary artery systolic pressure (PASP) > 50 mmHg, a surrogate of elevated left ventricular filling pressure. These findings support the concept that sonographic pulmonary congestion parallels biochemical and invasive markers of hemodynamic burden.

The composite event rate observed in our cohort was higher than that reported in major heart failure registries representing Asian, European, and global populations, including ASIAN-HF (12), ESC-HF-LT (13), and REPORT-HF (7). In these studies, composite outcome rates ranged from approximately 20–23.6% over 12 months of follow-up, whereas our cohort demonstrated a substantially higher event rate of 34.4% within a shorter follow-up duration of 6 months. When interpreted in the context of time-to-event exposure, this suggests a greater short-term event burden in our population. This difference is likely attributable to variations in the enrolled patient profiles. Both ASIAN-HF and ESC-HF-LT included a broader spectrum of heart failure patients, encompassing those with chronic and relatively stable disease, which may have attenuated overall event rates. In contrast, our study predominantly enrolled higher-risk patients, likely in the vulnerable post-acute or recently decompensated phase.

Our Cox proportional hazards analysis is likewise concordant with previous investigations evaluating the prognostic utility of LUS as a reference standard for congestion assessment (14). A meta-analysis by Suhardi et al. (15) demonstrated that residual B-lines were independently associated with a higher risk of composite clinical outcomes, including all-cause mortality and heart failure–related rehospitalization or cardiovascular events. Importantly, the risk exhibited a dose–response relationship, increasing proportionally with the number of residual B-lines detected.

However, in contrast to the findings reported by Mercedes Rivas-Lasarte et al. (2), in which lung ultrasound–detected subclinical pulmonary congestion remained independently associated with adverse outcomes after propensity score adjustment, the prognostic effect of NAVI-HF in our cohort attenuated following inclusion of established markers of heart failure severity, particularly NT-proBNP and LVEF. Importantly, NT-proBNP remained a strong and consistent predictor across all evaluated outcomes, underscoring its robust and well-established prognostic role in HF populations. This finding suggests that NAVI-HF may identify a higher-risk congestion phenotype, although its incremental prognostic value beyond established risk markers remains to be determined. Given that NT-proBNP reflects hemodynamic stress and congestion (6)—pathophysiological processes closely aligned with those detected by NAVI-HF—these variables likely represent parallel downstream manifestations of the same underlying disease severity. Therefore, adjustment for NT-proBNP does not primarily address classical confounding but rather assesses whether NAVI-HF provides incremental prognostic information beyond a dominant and established biomarker.

Finally, NAVI-HF was aimed for detecting clinical or subclinical pulmonary congestion, rather than replacing established prognostic biomarkers such as NT-proBNP. The objective of this phase was not to demonstrate superiority or complete independence from NT-proBNP, but to establish that NAVI-HF–detected congestion is clinically meaningful and associated with adverse outcomes. The attenuation observed in the fully adjusted model likely reflects overlapping pathophysiological information among congestion-related markers rather than an absence of prognostic relevance.

Although the present study evaluated patients in the predischarge phase, our results should be interpreted within this broader evidentiary framework. Given that LUS—serving as the reference standard for NAVI-HF—has established prognostic value in outpatient care (5, 16), it is biologically and clinically plausible that a simplified device such as NAVI-HF could offer similar risk stratification utility when applied beyond hospitalization.

Importantly, NAVI-HF was designed as an easy-to-use, training-independent tool suitable for home monitoring. The integration of such a device into routine heart failure management may facilitate earlier detection of recurrent or subclinical congestion, potentially enabling timely therapeutic adjustment. This concept is supported by meta-analytic evidence (17) demonstrating that LUS-guided therapy reduces the risk of major adverse cardiovascular events and heart failure rehospitalization compared with standard care. Subsequent studies (18, 19) published after that meta-analysis have further reinforced the promise of congestion-guided strategies.

Because NAVI-HF is conceptually and methodologically derived from LUS principles, it is reasonable to hypothesize comparable clinical utility in guiding decongestive management. Nevertheless, dedicated prospective trials evaluating NAVI-HF–guided therapy in outpatient and home-based settings are warranted to confirm this potential and to establish its incremental benefit over existing strategies.

Several limitations merit consideration. First, the relatively limited number of outcome events may reduce statistical power and increase the risk of overfitting in the multivariable Cox regression models, particularly in the fully adjusted analyses. Second, although NAVI-HF is intended for home-based monitoring in chronic heart failure populations, the present study was conducted in predischarge patients following acute decompensation. Therefore, extrapolation of these findings to stable ambulatory patients should be undertaken with caution. Third, lung ultrasound was used as the reference standard for congestion assessment due to its practicality and established clinical validation; however, invasive hemodynamic measurements were not performed in this phase. As a result, direct correlation with gold-standard parameters such as pulmonary capillary wedge pressure or right atrial pressure was not available, which may limit mechanistic interpretation of the findings. Lastly, our analysis considered only the first HF hospitalization and censored subsequent events, thereby evaluating time to first event rather than total hospitalization burden. Measuring recurrent hospitalizations over the 6-month period could have provided additional prognostic insight.

## Conclusion

In this second phase of the NAVI-HF study, positive NAVI-HF in predischarge heart failure patients was associated with an increased risk of adverse clinical outcomes particularly HF hospitalizations. These findings extend the initial diagnostic validation and demonstrate that NAVI-HF captures a clinically meaningful congestion phenotype with prognostic relevance.

## Data Availability

The original contributions presented in the study are included in the article/Supplementary Material, further inquiries can be directed to the corresponding author/s.
