# Supplementary material for: Clinical outcome implications of novel auscultation device of artificial intelligence (NAVI-HF) in predischarge acute heart failure patients
Source: Front Cardiovasc Med. 2026 Oct 27;13:1864004. doi: 10.3389/fcvm.2026.1864004 (PMC13748218; doi:10.3389/fcvm.2026.1864004)
Supplement: Supplementary file 1 [file Datasheet1.docx]

**Supplementary 1.** Sensitivity analysis of multiple Cox regression multivariate models

Composite Outcome

| **Variable** | **Sig.** | **Exp(B)** | **CI Lower** | **CI Upper** |
| --- | --- | --- | --- | --- |
| NAVI-HF | 0.627 | 1.124 | 0.701 | 1.804 |
| Age (per 1 year) | 0.581 | 1.006 | 0.984 | 1.029 |
| Gender | 0.265 | 0.739 | 0.435 | 1.257 |
| Hypertension | 0.464 | 1.196 | 0.741 | 1.93 |
| Diabetes | 0.213 | 1.335 | 0.848 | 2.102 |
| CKD | 0.565 | 1.165 | 0.692 | 1.962 |
| NTproBNP (per 100 unit) | 0.033 | 1.001 | 1 | 1.002 |
| LVEF (per 10 unit) | 0.001 | 0.635 | 0.481 | 0.837 |
| CAD | 0.166 | 0.674 | 0.386 | 1.179 |

| **Variable** | **Sig.** | **Exp(B)** | **CI Lower** | **CI Upper** |
| --- | --- | --- | --- | --- |
| NAVI-HF | 0.387 | 0.795 | 0.472 | 1.338 |
| Age (per 1 year) | 0.928 | 0.999 | 0.977 | 1.021 |
| Gender | 0.099 | 0.615 | 0.345 | 1.095 |
| Hypertension | 0.507 | 1.181 | 0.722 | 1.931 |
| Diabetes | 0.218 | 1.354 | 0.836 | 2.191 |
| CKD | 0.913 | 0.971 | 0.572 | 1.649 |
| LVEF (per 10 unit) | 0.051 | 0.729 | 0.531 | 1.001 |
| lnNTproBNP | <0.001 | 1.511 | 1.221 | 1.871 |

*LnNTpro BNP = per 1-unit increase in ln(NT-proBNP)*

| **Variable** | **Sig.** | **Exp(B)** | **CI Lower** | **CI Upper** |
| --- | --- | --- | --- | --- |
| NAVI-HF | 0.387 | 0.795 | 0.472 | 1.338 |
| Age (per 1 year) | 0.928 | 0.999 | 0.977 | 1.021 |
| Gender | 0.099 | 0.615 | 0.345 | 1.095 |
| Hypertension | 0.507 | 1.181 | 0.722 | 1.931 |
| Diabetes | 0.218 | 1.354 | 0.836 | 2.191 |
| CKD | 0.913 | 0.971 | 0.572 | 1.649 |
| LVEF (per 10 unit) | 0.051 | 0.729 | 0.531 | 1.001 |
| Log2NTproBNP | <0.001 | 1.331 | 1.148 | 1.544 |

*Log2 NTproBNP = per each doubling of NT-proBNP*

| **Variable** | **Sig.** | **Exp(B)** | **CI Lower** | **CI Upper** |
| --- | --- | --- | --- | --- |
| NAVI-HF | 0.031 | 1.668 | 1.049 | 2.652 |
| Age (per 1 year) | 0.681 | 0.996 | 0.974 | 1.017 |
| Gender | 0.167 | 0.677 | 0.39 | 1.177 |
| SBP | 0.115 | 1.015 | 0.996 | 1.035 |
| DBP | 0.082 | 0.976 | 0.949 | 1.003 |
| HR | 0.185 | 1.012 | 0.994 | 1.031 |

| **Variable** | **Sig.** | **Exp(B)** | **CI Lower** | **CI Upper** |
| --- | --- | --- | --- | --- |
| NAVI-HF | 0.14 | 1.477 | 0.88 | 2.477 |
| Age (per 1 year) | 0.883 | 0.998 | 0.975 | 1.022 |
| Gender | 0.337 | 0.731 | 0.385 | 1.387 |
| LVEF | 0.067 | 0.969 | 0.936 | 1.002 |
| LA diameter | 0.022 | 1.038 | 1.005 | 1.071 |
| E/A | 0.876 | 1.02 | 0.795 | 1.309 |
| E/e' | 0.588 | 1.007 | 0.981 | 1.034 |

| **Variable** | **Sig.** | **Exp(B)** | **CI Lower** | **CI Upper** |
| --- | --- | --- | --- | --- |
| NAVI-HF | 0.087 | 1.484 | 0.944 | 2.334 |
| Age (per 1 year) | 0.983 | 1 | 0.979 | 1.022 |
| Gender | 0.469 | 0.823 | 0.485 | 1.395 |
| ARNI | 0.407 | 0.68 | 0.272 | 1.695 |
| ACE/ARB | 0.916 | 0.954 | 0.397 | 2.29 |
| BB | 0.066 | 1.644 | 0.968 | 2.794 |
| MRA | 0.18 | 1.428 | 0.849 | 2.403 |
| SGLT2i | 0.657 | 0.81 | 0.32 | 2.049 |

| **Variable** | **Sig.** | **Exp(B)** | **CI Lower** | **CI Upper** |
| --- | --- | --- | --- | --- |
| NAVI-HF | 0.344 | 1.252 | 0.786 | 1.994 |
| Age (per 1 year) | 0.712 | 1.004 | 0.983 | 1.025 |
| Gender | 0.34 | 0.774 | 0.456 | 1.311 |
| NTproBNP (per 100 unit) | 0.006 | 1.002 | 1 | 1.003 |
| LVEF (per 10 unit) | 0.004 | 0.665 | 0.505 | 0.876 |
| SumGDMT | 0.124 | 1.214 | 0.948 | 1.554 |

*Sum GDMT = number of prescribed GDMT (beta- blockers, MRA, RAS inhibitors, SGLT2 inhibitors)*

HF Hospitalization

| **Variable** | **Sig.** | **Exp(B)** | **CI Lower** | **CI Upper** |
| --- | --- | --- | --- | --- |
| NAVI-HF | 0.526 | 1.169 | 0.722 | 1.891 |
| Age (per 1 year) | 0.724 | 1.004 | 0.982 | 1.027 |
| Gender | 0.285 | 0.742 | 0.43 | 1.282 |
| Hypertension | 0.225 | 1.361 | 0.827 | 2.238 |
| Diabetes | 0.307 | 1.27 | 0.803 | 2.01 |
| CKD | 0.458 | 1.217 | 0.724 | 2.047 |
| LVEF (per 10 unit) | <0.001 | 0.608 | 0.46 | 0.806 |
| NTproBNP (per 100 unit) | 0.115 | 1.001 | 1 | 1.002 |
| CAD | 0.13 | 0.647 | 0.369 | 1.136 |

| **Variable** | **Sig.** | **Exp(B)** | **CI Lower** | **CI Upper** |
| --- | --- | --- | --- | --- |
| NAVI-HF | 0.506 | 0.834 | 0.489 | 1.423 |
| Age (per 1 year) | 0.757 | 0.996 | 0.974 | 1.019 |
| Gender | 0.142 | 0.641 | 0.355 | 1.159 |
| Hypertension | 0.274 | 1.331 | 0.797 | 2.224 |
| Diabetes | 0.309 | 1.289 | 0.791 | 2.1 |
| CKD | 0.976 | 1.008 | 0.591 | 1.721 |
| LVEF (per 10 unit) | 0.028 | 0.692 | 0.499 | 0.961 |
| lnNTproBNP | <0.001 | 1.439 | 1.164 | 1.779 |

*LnNTpro BNP = per 1-unit increase in ln(NT-proBNP)*

| **Variable** | **Sig.** | **Exp(B)** | **CI Lower** | **CI Upper** |
| --- | --- | --- | --- | --- |
| NAVI-HF | 0.506 | 0.834 | 0.489 | 1.423 |
| Age (per 1 year) | 0.757 | 0.996 | 0.974 | 1.019 |
| Gender | 0.142 | 0.641 | 0.355 | 1.159 |
| Hypertension | 0.274 | 1.331 | 0.797 | 2.224 |
| Diabetes | 0.309 | 1.289 | 0.791 | 2.1 |
| CKD | 0.976 | 1.008 | 0.591 | 1.721 |
| LVEF (per 10 unit) | 0.028 | 0.692 | 0.499 | 0.961 |
| Log2NTproBNP | <0.001 | 1.287 | 1.111 | 1.491 |

*Log2 NTproBNP = per each doubling of NT-proBNP*

| **Variable** | **Sig.** | **Exp(B)** | **CI Lower** | **CI Upper** |
| --- | --- | --- | --- | --- |
| NAVI-HF | 0.023 | 1.736 | 1.08 | 2.789 |
| Age (per 1 year) | 0.484 | 0.992 | 0.971 | 1.014 |
| Gender | 0.145 | 0.655 | 0.371 | 1.158 |
| SBP | 0.092 | 1.017 | 0.997 | 1.036 |
| DBP | 0.054 | 0.973 | 0.946 | 1 |
| HR | 0.14 | 1.014 | 0.996 | 1.032 |

| **Variable** | **Sig.** | **Exp(B)** | **CI Lower** | **CI Upper** |
| --- | --- | --- | --- | --- |
| NAVI-HF | 0.151 | 1.471 | 0.869 | 2.49 |
| Age (per 1 year) | 0.731 | 0.996 | 0.972 | 1.02 |
| Gender | 0.258 | 0.681 | 0.35 | 1.324 |
| LVEF | 0.039 | 0.964 | 0.931 | 0.998 |
| LA diameter | 0.251 | 1.024 | 0.983 | 1.066 |
| E/A | 0.964 | 0.994 | 0.769 | 1.285 |
| E/e' | 0.69 | 1.005 | 0.979 | 1.033 |

| **Variable** | **Sig.** | **Exp(B)** | **CI Lower** | **CI Upper** |
| --- | --- | --- | --- | --- |
| NAVI-HF | 0.056 | 1.571 | 0.988 | 2.496 |
| Age (per 1 year) | 0.777 | 0.997 | 0.975 | 1.019 |
| Gender | 0.434 | 0.806 | 0.469 | 1.385 |
| ARNI | 0.53 | 0.746 | 0.299 | 1.861 |
| ACE/ARB | 0.999 | 1.001 | 0.416 | 2.406 |
| BB | 0.095 | 1.578 | 0.924 | 2.694 |
| MRA | 0.29 | 1.327 | 0.786 | 2.239 |
| SGLT2i | 0.761 | 0.866 | 0.342 | 2.192 |

| **Variable** | **Sig.** | **Exp(B)** | **CI Lower** | **CI Upper** |
| --- | --- | --- | --- | --- |
| NAVI-HF | 0.271 | 1.306 | 0.812 | 2.101 |
| Age (per 1 year) | 0.936 | 1.001 | 0.98 | 1.023 |
| Gender | 0.379 | 0.785 | 0.457 | 1.348 |
| LVEF (per 10 unit) | 0.002 | 0.637 | 0.481 | 0.845 |
| NTproBNP (per 100 unit) | 0.046 | 1.001 | 1 | 1.002 |
| SumGDMT | 0.241 | 1.161 | 0.904 | 1.491 |

*Sum GDMT = number of prescribed GDMT (beta- blockers, MRA, RAS inhibitors, SGLT2 inhibitors)*
